# Clinical Outcome of Minor Salivary Gland Cancers in the Oral Cavity: A Comparative Analysis With Squamous Cell Carcinomas of the Oral Cavity

**DOI:** 10.3389/fonc.2020.00881

**Published:** 2020-06-03

**Authors:** Song I Park, Woori Park, Sungyong Choi, Yunjeong Jang, Hyunjin Kim, Seok-Hyung Kim, Jae Myoung Noh, Man Ki Chung, Young-Ik Son, Chung-Hwan Baek, Han-Sin Jeong

**Affiliations:** ^1^Department of Otorhinolaryngology - Head and Neck Surgery, Samsung Medical Center, Sungkyunkwan University School of Medicine, Seoul, South Korea; ^2^Department of Pathology, Samsung Medical Center, Sungkyunkwan University School of Medicine, Seoul, South Korea; ^3^Department of Radiation Oncology, Samsung Medical Center, Sungkyunkwan University School of Medicine, Seoul, South Korea

**Keywords:** minor salivary gland, neoplasms, oral cavity, surgery, radiation, outcomes

## Abstract

**Purpose:** Salivary gland cancer (SGC) in the oral cavity is not common and has been less studied in comparison with oral squamous cell carcinoma (SCC). This study aimed to identify the clinical characteristics and outcomes of SGC in the oral cavity compared with oral SCC.

**Methods:** The medical charts of the patients with SGC (*N* = 68) arising from minor salivary glands and SCC (*N* = 750) in the oral cavity between 1995 and 2017 were reviewed retrospectively. The clinical and pathological factors and treatment outcomes were compared to identify clinical differences between oral SGC and SCC in total cases and in tumor size and subsite (propensity score)-matched pairs (*N* = 68 in each group). In addition, pattern of local invasion was pathologically assessed in a subset of SGC and SCC tumors.

**Results:** Patients with SGC in the oral cavity showed >90% survival at 5 years. Most common pathologies of SGC were mucoepidermoid carcinoma (39.7%) and adenoid cystic carcinoma (35.3%), where high-grade tumors (including adenoid cystic carcinomas having solid components, grade 2 or 3) represented only 36.8%. Compared with oral SCC, surgery for SGC had narrow surgical safety margin. However, local control was very successful in SGC even with <5 mm or positive resection margin through surgery plus adjuvant radiation treatments or surgery alone for small low-grade tumors. Pathologic analysis revealed that the frequency of oral SGC with infiltrative tumor border was significantly lower than that of oral SCC (46.4 vs. 87.2%, *P* < 0.001).

**Conclusions:** SGC in the oral cavity represents relatively good prognosis and has a locally less aggressive pathology compared with oral SCC. Adjuvant radiation can be very effective to control minimal residual disease in oral SGC. Our study proposed that a different treatment strategy for oral SGC would be reasonable in comparison with oral SCC.

## Introduction

Oral cancer is the sixth most common cancer worldwide (1). While the most common malignant disease in the oral cavity is squamous cell carcinoma (SCC), other pathologic types of malignancy including salivary gland cancers (SGC) can also occur in the oral cavity (1, 2). SGC is relatively rare and comprises 1–6% of all head and neck cancers (3–7). It has heterogeneous types of pathology with diverse tumor biology (4, 5, 8). Therefore, the clinical courses, outcomes and prognosis of intraoral SCC and SGC can be different, although they share the same anatomical site. Regarding adjuvant treatment, concurrent chemoradiation is a standard treatment modality for high-risk oral SCC as a postoperative adjuvant treatment (9). In contrast, adjuvant concurrent chemoradiation is not validated yet for high-risk SGC (currently under clinical trial) (10), and postoperative radiation is still a standard of care as an adjuvant treatment for SGC (11). In terms of prognosis, 5-year survival rate for patients with SCC ranges from 40 to 63% (2), while that for SGC is 71.8–90.1% and is characterized by late recurrence (6, 12, 13).

As for resectable SCC and SGC in the oral cavity, surgery is the primary treatment option (11). According to the National Comprehensive Cancer Network (NCCN) guidelines, a surgical safety margin of 5 mm is recommended to lower recurrence in SCC (9). Because of disease rarity, it is unclear whether this cutoff value in resection is valid for oral SGC. Previously, we demonstrated that close surgical margin <5 mm in SGC of the major salivary gland was not a significant risk factor for recurrence and not a good determinant for adjuvant radiation, particularly in low-grade tumors (14). As oral SGC is a submucosal lesion, it seems difficult to define the clear boundary of tumors due to the anatomical complexity. Therefore, it is clinically important to evaluate the local microscopic invasion into the surrounding tissues in oral SGC, which determines the surgical extent and post-operative adjuvant radiation treatments.

In the first attempt to answer this clinical question in decision making of surgical extent, we tried to identify the clinical outcomes, treatment response, and pattern of local invasion of oral SGC in comparison with those of oral SCC in this study. Unlike most previous studies dealing with SGC solely, we conducted a comparative study of oral SCC and SGC with tumor size and subsite-matched pairs. Thus, this study will provide clinically relevant information in treatment decision for oral SGC and will capture the biological differences of SCC and SGC with the same anatomic site of oral cavity.

## Materials and Methods

### Study Population

This retrospective study was approved by the Institutional Review Board of Samsung Medical Center. We collected and reviewed the medical records of SGC and SCC cases in the oral cavity that had been diagnosed and managed in our facility from 1995 to 2017. The diagnoses were confirmed by pathology. The SGCs in the oral cavity originated from minor salivary glands in the oral cavity, and we excluded the cases from sublingual glands. A total of 818 patients (68 SGC and 750 SCC) were included in this study, after exclusion of cases with incomplete clinical information or undetermined pathology.

### Clinical and Pathological Variables

Clinical and pathological data of age, gender, site of tumor, tumor grade, tumor-node-metastasis (TNM) stage, surgical margin status, extranodal extension, type of treatment and treatment outcome were analyzed. The staging of all cases was based on the TNM classification of the American Joint Committee on Cancer (AJCC) staging manual (15). As for the cases included in this study, we reviewed the pathology again by experienced pathologists who has more than 10 years of experience in salivary gland pathology. The histological typing was made or revised according to the 2017 World Health Organization classification of salivary tumors (16). If two or more pathology types were mixed, the tumor was classified as the pathological type with the worst prognosis. The histological grade of tumor was defined as low, intermediate or high according to cytological features and architectures (16–18). Mucoepidermoid carcinomas were divided into 3 grades, based on the accepted criteria (17). Adenoid cystic carcinomas were graded according to the proportion of solid component; grade 1: predominantly tubular type with no solid component, grade 2: predominantly cribriform type with solid component less than 30%, grade 3: solid component more than 30% (17, 19). Adenocarcinoma were classified as high or low group by histological type and cytological variants (17). Acinic cell, clear cell and myoepithelial carcinoma were classified as low grade, while salivary duct carcinoma was classified as high grade (17).

### Treatments and Follow-Ups

Most patients were managed with initial surgery-based treatments for resectable disease. Surgery was intended to remove all cancer tissues in the primary site and neck lymph nodes. Neck dissection was conducted simultaneously for clinically suspicious (therapeutic) or occult (elective) lymph nodes in the neck, following the accepted surgical guidelines (NCCN guidelines). Surgical defects were reconstructed with a flap or local tissue, if indicated.

During the study period, radiation techniques were mainly three-dimensional conformal radiation or intensity-modulated radiation, with a mean dose of 61.0 Gy (range 50.0–70.0) by 2.0 or 2.2 Gy (mean 2.1 Gy, range 1.8–2.5) per fraction (mean 29.6 fractions, range 24–35) over 5.5–6 weeks.

For radiotherapy (RT) plan, patients underwent computed tomography (CT) scans with a thermoplastic mask. In adjuvant RT, clinical target volume (CTV) included the primary tumor bed and pathologically involved regional lymphatics with adequate margins. Elective neck irradiation (ENI) including the remote and uninvolved lymphatic levels was determined on an individual basis, considering the estimated risk of metastasis based on location, histologic type, extent, and grade of primary tumor. RT was delivered with 4- or 6-MV photons generated from a linear accelerator.

For patients receiving definitive RT, gross tumor volume (GTV) was defined as volume of primary tumor and involved lymph nodes based on all available clinical information. The CTV of primary tumor was delineated by adding 5 mm margins in all directions from GTV, and the margins were optionally modified in accordance with the anatomic boundaries of the tumor location and/or the adjacent organs.

Chemotherapy was administered concurrently with radiation in the adjuvant setting (oral SCC), or independently in the palliative setting. Cisplatin was the major drug for chemotherapy, in combination with other drugs depending on medical oncologist decision and clinical situation.

In terms of treatment outcome, recurrence was defined when suspicious lesions were apparent on imaging or confirmed by biopsy. The survival period was defined as the time from diagnosis to death of any cause.

### A Propensity Score-Matching Analysis

A propensity score-matching method was used between oral SGC and oral SCC groups to minimize differences in baseline characteristics by using JMP macro software (SAS Institute Inc., Cary, NC, USA). T status and subsites were included in the propensity matching model. Tumor subsites in the oral cavity were roughly divided into three subsites; tongue and floor of mouth (central soft tissues), hard palate and retromolar trigone (mucoperiosteal tissue), and lip and buccal area (lateral soft tissues).

Patients were matched at a 1:1 ratio using the caliper method (caliper width = 0.25 standard deviation). Finally, 136 cases (*N* = 68 in the oral SGC group and *N* = 68 in the oral SCC group) were allocated to the comparison groups (Supplementary Material). Comparisons between the two groups were performed by stratified Chi-square test for categorical variables.

### Pathologic Analysis

Under propensity score matching, 120 patients (out of 136) had been managed with surgical treatments alone or in combination with other treatment modalities. To evaluate the pathological pattern of the tumor border (the surgical margin between tumors and adjacent tissues), we excluded 19 tumor samples of positive cancer cells at the resection margin (where there was no adjacent normal tissues in the surgical margin) or unknown cases. Another 26 cases were excluded from pathologic analyses, because of unavailable or poor quality of surgical pathology tissues. Therefore, a total of 75 patient samples were included in the pathologic analysis (28 SGC and 47 SCC tumors). The status of resection margin (pathological local infiltration) included the presence of perineural invasion. If there were perineural invasion at the resection margin or less than 5 mm away from the resection margin, we regarded them as positive or close resection margin.

As for each tumor, multiple pathology slides (three to seven) were reviewed by two pathologists. Through pathology review, the tumor margin was classified as a pushing or infiltrative border. A pushing border was defined as cancer cells forming a single lump with a clear boundary. Meanwhile, an infiltrative border was defined as tumor cells penetrating into the surrounding matrix without linear demarcation between tumor and adjacent tissues (20–22). In equivocal cases, the joint decision was made by a consensus or discussion of two raters.

### Statistical Analysis

Propensity score analysis with 1:1 matching was used as previously described to match a cohort of patients with oral SGC to patients with oral SCC. All variables were examined using Fisher's exact test or Pearson chi-square test. Survival analysis was performed using Kaplan-Meier estimate and statistical significance was determined by log-rank test. The data were analyzed using the statistical package for social science (SPSS) (IBM Corporation, Armonk, NY, USA). Differences for *P*-value less than 0.05 were regarded as statistically significant.

## Results

### Comparison of Outcomes in a Pooled Cohort

The detailed characteristics of patients in the oral SGC (*N* = 68) and SCC (*N* = 750) groups are presented in Table 1. Female was significantly dominant in SGC compared to SCC. Hard palate/retromolar trigone and tongue/floor of the mouth were the most common origin of SGC and SCC in the oral cavity, respectively. The most common pathology type in SGC was mucoepidermoid carcinoma (39.7%), followed by adenoid cystic carcinoma and adenocarcinoma not otherwise specified (Table 2). Unlike SCC, low grade tumor (excluding adenoid cystic carcinomas) was the most common tumor grade in oral SGC, comprising 54.4%.

**Table 1:** Comparison of total subjects diagnosed with minor salivary gland origin cancer or mucosal squamous cell carcinomas of the oral cavity (Salivary gland cancer *N* = 68, oral squamous cell carcinoma *N* = 750).

**No. (%)**	**Salivary gland cancer (*N* = 68)**	**Squamous cell carcinoma (*N* = 750)**	**Difference (*P*)**
Age (median, range, years)	51.0 (23.0–86.0)	56.0 (18.0–97.0)	0.074
Gender (Male: Female)	26:42 (38.2:61.8)	473:277 (63.1:36.9)	**<0.001**
Tumor subsite			**<0.001**
Hard palate/ Retromolar trigone	41 (60.3)	70 (9.3)	
Tongue/ Floor of the mouth	17 (25.0)	580 (77.3)	
Buccal/ Lip	10 (14.7)	100 (13.3)	
TNM status			
T T1	23 (33.8)	319 (42.5)	**0.028**
T2	24 (35.3)	238 (31.7)	
T3	5 (7.4)	76 (10.1)	
T4	16 (23.5)	87 (11.6)	
Tx	0 (0.0)	30 (4.0)	
N N0	55 (80.9)	461 (61.5)	**<0.001**
N1	3 (4.4)	75 (10.0)	
N2	3 (4.4)	177 (23.6)	
N3	7 (10.3)	11 (1.5)	
Nx	0	26 (3.5)	
M M1 at presentation	4 (5.9)	5 (0.7)	**0.004**
TNM stage			0.078
I	22 (32.4)	271 (36.1)	
II	16 (23.5)	133 (17.7)	
III	4 (5.9)	95 (12.7)	
IV	26 (38.2)	220 (29.3)	
Unknown	0	31 (4.1)	
Extranodal extension	6 (9.8)	128 (18.6)	0.055
Treatments			**<0.001**
Surgery alone	25 (36.8)	363 (48.4)	
Surgery + radiation	36 (52.9)	200 (26.7)	
Surgery + chemoradiation	0 (0.0)	126 (16.8)	
Chemoradiation	3 (4.4)	33 (4.4)	
Radiation alone	3 (4.4)	21 (2.8)	
Chemotherapy	0 (0.0)	5 (0.7)	
No treatments^a^	1 (1.5)	2 (0.3)	
Status of resection margin in cases of surgery	*N* = 61	*N* = 689	**<0.001**
Safety margin ≥ 5 mm	11 (18.0)	338 (49.1)	
Close margin <5 mm	33 (54.1)	296 (43.0)	
Positive cancer cells at resection	13 (21.3)	27 (3.9)	
margin			
Unknown	4 (6.6)	28 (4.1)	
Treatment outcomes (No. %) and event time points (median, [interquartile range], months)			
No evidence of disease at the last	42 (61.8)	424 (56.5)	0.444
follow up			
Local recurrence^b^	5 (7.4) (16.0 [13.4–52.3])	122 (16.3) (8.5 [4.3–27.0])	0.054
Regional recurrence^b^	4 (5.9) (43.1 [33.5–49.9])	123 (16.4) (6.4 [3.9–9.9])	**0.022**
Distant metastasis^b^	19 (27.9) (41.1 [10.8–58.5])	44 (5.9) (6.3 [3.9–11.8])	**<0.001**
Disease progression^c^	0	38 (5.1) (2.8 [1.2–4.4])	0.066
Death of disease	9 (13.2) (46.0 [38.1–89.9])	195 (26.0) (17.3 [10.7–33.6])	**0.019**
Unknown	2 (2.9)	15 (2.0)	0.645
Follow-up (median, range, months)	49.9 (0.9–245.8)	31.3 (0.7–235.3)	

a *No cancer treatments due to acute tumor bleeding, poor medical condition or refusal of recommended treatment*.

b *Number overlapped*.

c *Disease progression on non-surgical treatment*.

*Bold P-values indicate P < 0.05*.

**Table 2:** Pathology diagnosis of enrolled subjects.

**No. (%)**	**Salivary gland cancer (*N* = 68)**
Pathology and tumor grade	
Mucoepidermoid carcinoma	27 (39.7)
High/ Intermediate/ Low	4/ 1/ 22 (14.8/ 3.7/ 81.5)
Adenoid cystic carcinoma^a^	24 (35.3)
Grade 3/ Grade 2/ Grade 1	3/ 16/ 5 (12.5/ 66.7/ 20.8)
Adenocarcinoma, NOS	11 (16.2)
High/ Intermediate/ Low	1/ 0/ 10 (9.1/ 0/ 90.9)
Acinic cell carcinoma (low grade)	2 (2.9)
Salivary duct carcinoma (high grade)	1 (1.5)
Clear cell carcinoma (low grade)	1 (1.5)
Myoepithelial carcinoma (low grade)	2 (2.9)

*Adenocarcinoma, NOS: Adenocarcinoma, not otherwise specified*.

a *Grade 1: predominantly tubular type, no solid component), grade 2: predominantly cribriform type, solid component equal to or less than 30%, and grade 3: solid component more than 30%*.

Regarding T and N status, there was a higher T tendency for oral SGC and higher N status for oral SCC, which were similar to the previous report (23). The percentage of M1 was higher in SGC group, mainly due to the adenoid cystic carcinoma pathology. Pre-operative histopathological diagnosis was made by fine needle aspiration cytology or biopsy which was correct in 54.7% of surgical cases (29 out of 53 cases). Tumor grade was correctly predicted in 30.2% of cases (16 out of 53 cases) preoperatively.

**Figure 1:**
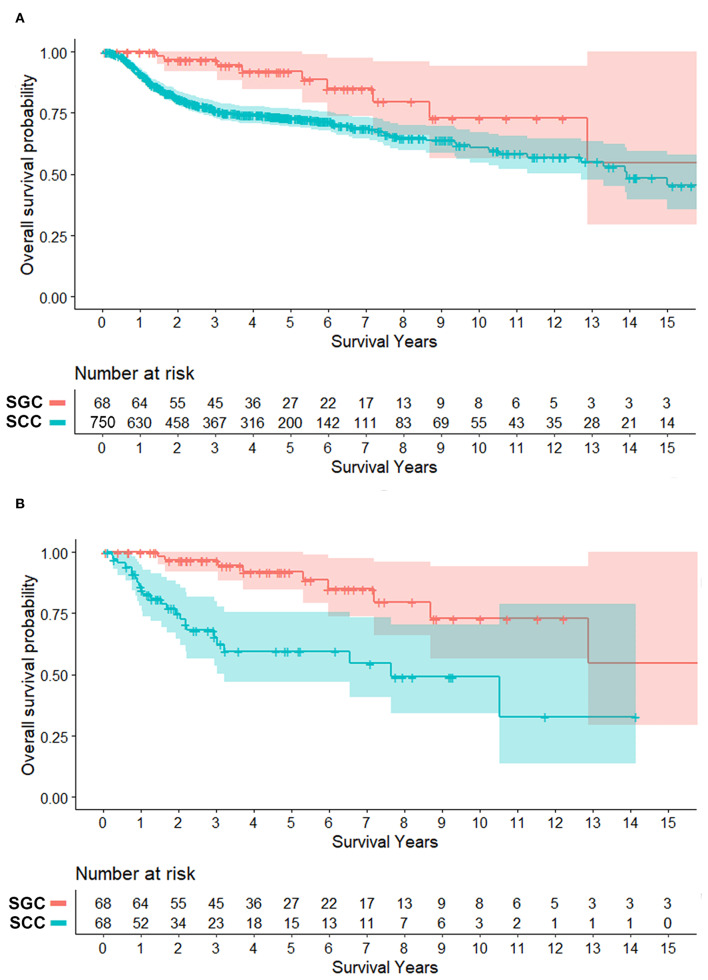
Survival plots of patients with minor salivary gland cancer (SGC) and mucosal squamous cell carcinoma (SCC) in the oral cavity (overall survival). **(A)** Total patients. **(B)** Patients matched for tumor size (T status) and subsite (propensity score, 1:1). Shaded area = 95% confidence interval. Overall survival rates at 5, 10, and 15 years for oral SGC were 91.9, 72.9, and 54.7%, respectively. Those for oral SCC were 73.2, 61.8% and 48.6% (*N* = 750) and 59.6, 49.1, and 32.8% (*N* = 68 in matched cases).

Surgery was the primary treatment option in SCC, and surgery with adjuvant radiation (52.9%) was the main treatment for SGC. Adjuvant chemoradiation was only performed in oral SCC, but not SGC, in our institute. Notably in patients with surgery, half of SCC group (49.1%) had more than 5 mm of resection margin; meanwhile a larger proportion of patients (75.4%) had close or positive resection margin in the SGC group. In clinical courses, regional recurrence and disease-related deaths were more frequent in SCC, while distant metastasis more commonly occurred in SGC. The overall survival difference between the two groups was significant (Figure 1A). The 5-year overall survival rate was 91.9% in SGC and 73.2% in SCC, respectively (*P* = 0.0015). Ten- and fifteen-year overall survival rates for oral SGC were 72.9 and 54.7%, and those for oral SCC were 61.8 and 48.6%, respectively.

### Outcome Comparison in a Propensity Score-Matched Cohort

In this study, we focused on local tumor control and local extension (pathological infiltration) of oral SGC and SCC. Thus, we adjusted three potential factors in comparison between oral SGC and SCC: T status, tumor subsites, and tumor grade.

First, we constructed a propensity score-matched cohort, using T status and tumor subsite. In this propensity score matching, we tried a matching between two groups with various ratios (1:1, 1:2, 1:3) and caliper widths (0.05, 0.1, 0.2, 0.25), but the matching outcomes were suboptimal (standard mean differences in matching variables > 0.1) except 1:1 matching and caliper width = 0.25. Thus, patients with SCC (*N* = 68) were matched with 68 patients diagnosed with SGC at a 1:1 ratio using the caliper method (caliper width = 0.25 standard deviation). The result of a propensity-score matching was satisfactory according to the T status and tumor subsite (Table 3).

**Table 3:** Results of propensity score-matching (1:1) using a caliper of 0.25.

	**Before propensity score matching**			**After a propensity score-matching**		
Standardized mean difference in matching variables						
Subsite		1.543			<0.001	
T status		0.025			0.029	
Matching variables (No. %)						
	**SGC (*****N*** **=** **68)**	**SCC (*****N*** **=** **750)**	***P*****-value**	**SGC (*****N*** **=** **68)**	**SCC (*****N*** **=** **68)**	***P*****-value**
Subsite			<0.001			>0.999
Hard palate/ Retromolar trigone	41 (60.3)	70 (9.3)		41 (60.3)	41 (60.3)	
Tongue/ Floor of the mouth	17 (25.0)	580 (77.3)		17 (25.0)	17 (25.0)	
Buccal/ Lip	10 (14.7)	100 (13.3)		10 (14.7)	10 (14.7)	
T status			0.072			>0.999
T1–2	47 (69.1)	557 (74.3)		47 (69.1)	47 (69.1)	
T3–4	21 (30.9)	163 (21.7)		21 (30.9)	21 (30.9)	
Tx		30 (4.0)				

After propensity score matching (Table 4), oral SGC and SCC groups had unique features regarding gender distribution (female predominance in SGC), nodal metastasis, extranodal extension and treatment types. Similarly to those of a pooled cohort, surgical safety margin more than 5 mm was more frequent in the oral SCC group (18.0% in SGC vs. 33.9% in SCC), and the rate of presence of cancer cells at the resection margin was higher in SGC than in SCC (21.3 vs. 3.4%) (*P* = 0.001).

**Table 4:** Clinical characteristics of tumor size (T status) and subsite (propensity score, 1:1)-matched salivary gland cancer (*N* = 68) and squamous cell carcinoma (*N* = 68) in the oral cavity.

**No. (%)**		**Salivary gland cancer (*N* = 68)**	**Squamous cell carcinoma (*N* = 68)**	**Difference (*P*)**
Age (median, range, years)		52.8 (23.0–86.0)	59.8 (34.0–82.0)	**0.003**
Gender (Male: Female)		26:42 (38.2:61.8)	47:21 (69.1:30.9)	**0.001**
Tumor subsite				>0.999
	Hard palate/ Retromolar trigone	41 (60.3)	41 (60.3)	
	Tongue/ Floor of the mouth	17 (25.0)	17 (25.0)	
	Buccal/ Lip	10 (14.7)	10 (14.7)	
TNM status				
T	T1–2	47 (69.1)	47 (69.1)	>0.999
	T3–4	21 (30.9)	21 (30.9)	
N	N0	55 (80.9)	37 (54.4)	**0.002**
	N1–3	13 (19.1)	30 (44.1)	
	Nx	0 (0.0)	1 (1.5)	
M	M1 at presentation	4 (5.9)	0 (0.0)	0.119
TNM stage				0.271
	I	22 (32.4)	21 (30.9)	
	II	16 (23.5)	8 (11.8)	
	III	4 (5.9)	7 (10.3)	
	IV	26 (38.2)	31 (45.6)	
	Unknown	0 (0.0)	1 (1.5)	
Extranodal extension		6 (9.8)	16 (27.1)	**0.018**
Treatments				**<0.001**
	Surgery alone	25 (36.8)	29 (42.6)	
	Surgery + radiation	36 (52.9)	15 (22.1)	
	Surgery + chemoradiation	0 (0.0)	15 (22.1)	
	Chemoradiation	3 (4.4)	6 (8.8)	
	Radiation alone	3 (4.4)	3 (4.4)	
	No treatment^a^	1 (1.5)	0 (0.0)	
Status of resection margin in cases of surgery		*N* = 61	*N* = 59	**0.001**
	Safety margin ≥ 5 mm	11 (18.0)	20 (33.9)	
	Close margin <5 mm	33 (54.1)	37 (62.7)	
	Positive cancer cells at resection margin	13 (21.3)	2 (3.4)	
	Unknown	4 (6.6)	0 (0.0)	
Treatment outcomes (No. %) and event time points (median, [interquartile range], months)				
	No evidence of disease at the last follow up	42 (61.8)	34 (50.0)	0.227
	Local recurrence^b^	5 (7.4) (16.0 [13.4–52.3])	14 (20.6) (6.4 [3.5–11.6])	**0.046**
	Regional recurrence^b^	4 (5.9) (43.1 [33.5–49.9])	14 (20.6) (7.3 [3.1–11.7])	**0.021**
	Distant metastasis^b^	19 (27.9) (41.1 [10.8–58.5])	4 (5.9) (1.5 [1.1–8.8])	**0.001**
	Disease progression^c^	0	4 (5.9) (3.7 [3.2–4.7])	0.119
	Death of disease	9 (13.2) (46.0 [38.1–89.9])	24 (35.3) (17.4 [10.5–30.0])	**0.005**
	Unknown	0	2 (2.9)	0.496
Follow-ups (median, range, months)		49.9 (0.9–245.8)	24.4 (0.7–176.9)	

a *Poor medical condition*.

b *Number overlapped*.

c *Disease progression on non-surgical treatment*.

*Bold P-values indicate P < 0.05*.

In clinical course, local and regional recurrence rates were higher in SCC even with wider resection of SCC, but distant metastasis was detected frequently in SGC (27.9 vs. 5.9%, *P* = 0.001). The overall survival plot was also similar to that of a pooled cohort. The 5-year overall survival rates were 91.9 and 59.6% (*P* < 0.001) (Figure 1B). Ten- and fifteen-year overall survival rates for oral SGC were 72.9 and 54.7%, and those for oral SCC were 49.1 and 32.8%, respectively.

### Response to Treatment and Pattern of Failure

Considering better oncological outcomes even with high rate of marginal surgical resection in SGC (close or positive resection margin), we evaluated the potential effectiveness of adjuvant treatments in cases with close or positive resection margin in surgical specimens (Table 5).

**Table 5:** Response to treatments and pattern of failures in cases with close or positive resection margins (Salivary gland cancer *N* = 46, Squamous cell carcinoma *N* = 39).

**No. (%)**		**Salivary gland cancer (*N* = 46)**	**Squamous cell carcinoma (*N* = 39)**	**Difference (*P*)**
Close resection margin (<5 mm in surgical safety margin)		*N* = 33	*N* = 37	
No adjuvant radiation		11 (33.3)^a^	17 (45.9)^b^	**<0.001**
Adjuvant radiation		22 (66.7)	9 (24.3)^c^	
Adjuvant chemoradiation		0	11 (29.7)	
Treatment outcomes (No. %) and event time points (median, [interquartile range], months)				
Local control		33 (100.0)	26 (70.3)	**0.001**
Local recurrence		0	8 (21.6) (9.1 [2.9–16.1])	
Unknown at primary site		0	3 (8.1)	
Regional recurrence^d^		1 (3.0) (41.1)	8 (21.6) (7.4 [3.5–13.7])	**0.03**
Distant metastasis^d^		10 (30.3) (32.3 [13.4–44.7])	3 (8.1) (1.7 [1.5–15.8])	**0.029**
Positive cancer cells at resection margin		*N* = 13	*N* = 2	
No adjuvant radiation		3 (23.1)^e^	1 (50.0)^b^	0.476
Adjuvant radiation		10 (76.9)	1 (50.0)^c^	
Adjuvant chemoradiation		0	0	
Treatment outcomes (No. %) and event time points (median, [interquartile range], months)				
Local control		10 (76.9)	0	**0.038**
Local recurrence		1 (7.7) (83.7)	2 (100.0) (20.6 [12.7–18.6])	
Unknown at primary site		2 (15.4)	0	
Regional recurrence^d^		1 (7.7) (10.8)	2 (100.0) (20.6 [12.7–18.6])	**0.029**
Distant metastasis^d^		5 (38.5) (59.5 [10.8–83.7])	0	0.524
Follow-ups (median, range, months)		45.8 (0.9–152.9)	26.0 (0.7–146.8)	

a *Cases with low-grade salivary gland cancer and small tumor burden*.

b *Cases with small tumor burden without pathologic risk factors, reluctant to undergo radiation treatment, or occurrence of systemic metastasis*.

c *Poor patient performance status for concurrent chemo-radiation*.

d *Number overlapped*.

e *Loss of follow-up loss in 1 patient and clinical follow-up only due to systemic disease in 1 patient*.

*Bold P-values indicate P < 0.05*.

When comparing the two groups with close resection margin according to treatment type and clinical outcomes, the SGC group had more adjuvant radiation (66.7 vs. 24.3%, *P* < 0.001), and higher local control rate (100.0 vs. 70.3%, *P* = 0.001), lower regional recurrence rate (3.0 vs. 21.6%, *P* = 0.03) and higher distant metastasis rate (30.3 vs. 8.1%, *P* = 0.029). This suggested that adjuvant radiation may play an essential role in local control of oral SGC. Even in cases with positive resection margin, adjuvant radiation successfully achieved local control in oral SGC (76.9 vs. 0.0%, *P* = 0.038).

Another interesting finding was that there was just one local recurrence even with surgery alone (without any adjuvant treatment) for oral SGC with marginal resection surgery (Table 5). These tumors were usually small and low grade tumors. Thus, it appeared that a surgery of <5 mm safety margin would be acceptable for low-risk oral SGC tumors.

### Subgroup Analysis With Tumor Grade Adjustment

In the initial subjects, the number of high-grade tumors (including grade 2 or 3 adenoid cystic carcinomas) in oral cavity SGC was not big enough (*N* = 25 of 68, 36.8%). Thus, we could not include tumor grade as a variable in a propensity score matching. Rather, we adjusted tumor grade in this subgroup comparison.

To understand the effect of tumor grade on local tumor control, we only included in a subset with high grade tumors from a previous propensity score-matched cohort (Table 6). In this analysis, we included adenoid cystic carcinoma cases, because they are locally aggressive (infiltrative) regardless of grade (18). Similarly, use of adjuvant radiation (not chemoradiation) (63.3 vs. 22.1%, *P* < 0.001) and safety resection margin ≥ 5 mm (8.3 vs. 33.9%, *P* < 0.001) were different between SGC and SCC groups. In patients with high grade SGC, systemic spread occurred in the clinical course in 33.3% (5.9% in SCC). In line with the previous findings, adjuvant radiation treatment in close or positive resection margin even in high-grade SGC appeared to be very effective in terms of local control (Table 7).

**Table 6:** Comparison of tumor size, subsite, and tumor grade-matched salivary gland cancer (high-grade, including adenoid cystic carcinoma) (*N* = 30) and squamous cell carcinoma (*N* = 68) in the oral cavity.

**No. (%)**		**High-grade salivary gland cancer (*N* = 30)**	**Squamous cell carcinoma (*N* = 68)**	**Difference (*P*)**
Age (mean, range, years)		58.3 (28.0–82.0)	59.8 (34.0–82.0)	0.596
Gender (Male: Female)		9:21 (30.0:70.0)	47:21 (69.1:30.9)	**<0.001**
Pathology				
	Squamous cell carcinoma		68 (100.0)	**<0.001**
	Mucoepidermoid carcinoma	4 (13.3)		
	Adenocarcinoma, NOS	1 (3.3)		
	Salivary duct carcinoma	1 (3.3)		
	Adenoid cystic carcinoma	24 (80.0)		
Tumor subsite				0.555
	Hard palate/ Retromolar trigone	15 (50.0)	41 (60.3)	
	Tongue/ Floor of the mouth	11 (36.7)	17 (25.0)	
	Buccal/ Lip	4 (13.3)	10 (14.7)	
TNM status				
T	T1–2	16 (53.3)	47 (69.1)	0.171
	T3–4	14 (46.7)	21 (30.9)	
N	N0	21 (70.0)	37 (54.4)	0.330
	N1–3	9 (30.0)	30 (44.1)	
	Nx	0	1 (1.5)	
M	M1 at presentation	4 (13.3)	0 (0.0)	**0.008**
TNM stage				**0.004**
	I	1 (3.3)	21 (30.9)	
	II	9 (30.0)	8 (11.8)	
	III	2 (6.7)	7 (10.3)	
	IV	18 (60.0)	31 (45.6)	
	Unknown	0	1 (1.5)	
Extranodal extension		3 (12.5)	16 (27.1)	0.248
Treatments				**<0.001**
	Surgery alone	5 (16.7)	29 (42.6)	
	Surgery + radiation	19 (63.3)	15 (22.1)	
	Surgery + chemoradiation	0	15 (22.1)	
	Chemoradiation	3 (10.0)	6 (8.8)	
	Radiation alone	2 (6.7)	3 (4.4)	
	No treatments	1 (3.3)	0	
Status of resection margin in cases with surgery		*N* = 24	*N* = 59	**<0.001**
	Safety margin ≥ 5 mm	2 (8.3)	20 (33.9)	
	Close margin <5 mm	11 (45.8)	37 (62.7)	
	Positive cancer cells at resection margin	11 (45.8)	2 (3.4)	
Treatment outcomes (No. %) and event time points (median, [interquartile range], months)				
	No evidence of disease at the last follow-up	10 (13.5)	34 (50.0)	0.186
	Local recurrence^b^	1 (3.3) (83.6)	14 (20.6) (6.4 [3.5–11.6])	**0.033**
	Regional recurrence^b^	2 (6.7) (43.1 [42.1–44.2])	14 (20.6) (7.3 [3.4–11.7])	0.137
	Distant metastasis^b^	10 (33.3) (44.3 [27.9–59.8])	4 (5.9) (1.5 [1.1–8.8])	**0.001**
	Death of disease	5 (16.7) (18.7 [10.7–43.4])	24 (35.3) (17.4 [10.5–30.0])	0.092
	Unknown	2 (6.7)	2 (2.9)	0.584
Follow-ups (median, range, months)		53.3 (0.9–109.9)	24.4 (0.7–176.9)	

*Adenocarcinoma, NOS: Adenocarcinoma, not otherwise specified*.

b *Number overlapped*.

*Bold P-values indicate P < 0.05*.

**Table 7:** Response to treatments and pattern of failures in cases with close or positive resection margins in high-grade salivary gland cancer (including adenoid cystic carcinoma) (*N* = 22) and squamous cell carcinoma (*N* = 39).

**No. (%)**		**High-grade salivary gland cancer (*N* = 22)**	**Squamous cell carcinoma (*N* = 39)**	**Difference (*P*)**
Close resection margin		*N* = 11	*N* = 37	
No adjuvant radiation		1 (9.1)^a^	17 (45.9)^b^	**<0.001**
Adjuvant radiation		10 (90.9)	9 (24.3)^c^	
Adjuvant chemoradiation		0	11 (29.7)	
Treatment outcomes (No. %) and event time points (median, [interquartile range], months)				
Local control		11 (100.0)	26 (70.3)	**0.048**
Local recurrence^d^		0	8 (21.6) (9.1 [2.9–16.1])	0.170
Unknown at primary site		0	3 (8.1)	0.999
Regional recurrence^d^		1 (9.1) (41.1)	8 (21.6) (7.4 [3.5–13.7])	0.662
Distant metastasis^d^		7 (63.6) (41.1 [17.2–44.3])	3 (8.1) (1.5 [1.1–8.8])	**<0.001**
Positive cancer cells at resection margin		*N* = 11	*N* = 2	
No adjuvant radiation		3 (27.3)^a^	1 (50.0)^b^	0.999
Adjuvant radiation		8 (72.7)	1 (50.0)^c^	
Adjuvant chemoradiation		0	0	
Treatment outcomes (No. %) and event time points (median, [interquartile range], months)				
Local control		8 (72.7)	0 (0.0)	0.128
Local recurrence^d^		1 (9.1) (3.3)	2 (100.0) (4.5 [4.5–8.8])	**0.038**
Unknown at primary site		2 (18.2)	0	
Regional recurrence^d^		0	2 (100.0) (6.0 [6.0–8.0])	0.333
Distant metastasis^d^		3 (27.3)	0	
Follow-up (median, range, months)		44.5 (0.9–110.6)	25.9 (0.7–146.8)	

a *Cases with small tumor burden or reluctant to undergo adjuvant treatment*.

b *Cases with small tumor burden without pathology risk factors, reluctant to undergo radiation treatment, or occurrence of systemic metastasis*.

c *Poor patient performance status for concurrent chemo-radiation*.

d *Number overlapped*.

*Bold P-values indicate P < 0.05*.

### Pathologic Analysis of Microscopic Tumor Extension

**Figure 2:**
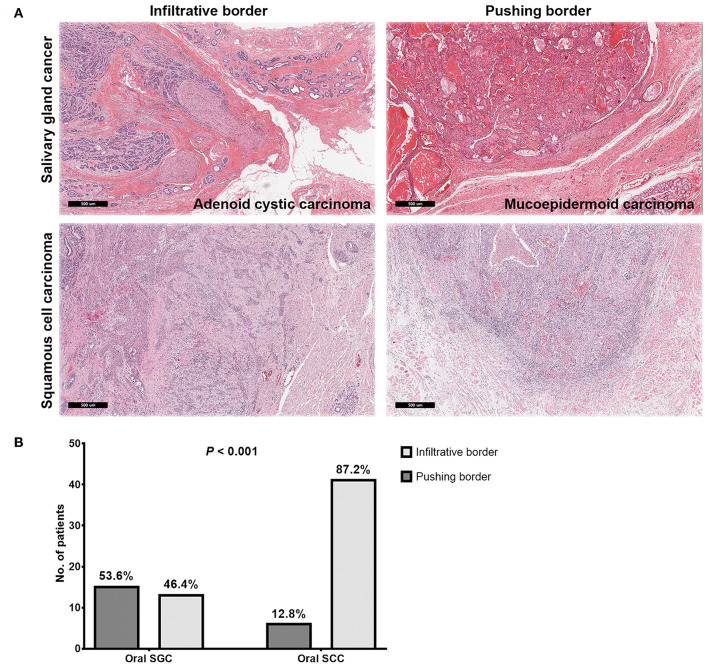
Representative images of microscopic invasion of tumors in salivary gland cancer and squamous cell carcinoma of the oral cavity **(A)** and comparison of tumor border pattern between oral salivary gland cancer and squamous cell carcinomas **(B)**.

Even with marginal surgical resection of oral cavity SGC, we found excellent local tumor control with surgery alone or surgery plus adjuvant radiation in our series, regardless of tumor grade. In addition to the effective role of adjuvant radiation, we compared microscopic tumor borders of oral SGC, with those of oral cavity SCC. Thus, we pathologically re-analyzed the surgical specimens (cases with close and clear resection margins) from a propensity-matched cohort. Remarkably, most SCC tumors had an infiltrative border (41 out of 47, 87.2%); while only 46.4% (13 out of 28) of SGC tumors had an infiltrative border (*P* < 0.001) (Figure 2). Thus, oral SGC had a locally less aggressive pathology, compared with oral SCC.

## Discussion

Most malignancies arising from the oral cavity are SCC, and SGC of the oral cavity is relatively rare (23). Therefore, determination of an optimal treatment strategy for oral SGC is difficult due to lack of sufficient evidence. For this reason, current treatment for oral SGC largely depends on clinical data from SCC of the oral cavity and of the head and neck (7, 24). However, it is not yet clear whether the current surgical treatment and indications for adjuvant treatment are suitable for treatment of oral SGC, even though these two types of carcinomas have different clinical and biological characteristics (25). In this study, we tried to identify the clinical and treatment characteristics of oral SGC, compared to oral SCC. There had been several studies investigating the clinical features of SGC arising from the oral cavity or oropharynx (23–32), but no comparative analysis of oral SGC and SCC has been published.

The mean age at diagnosis in our patients with oral SGC was 51.0 years, which is similar to those of other reports (6, 23, 27, 29, 30). In terms of male/female ratio, our female preponderance was also comparable with other studies with a ratio range from 1:1.2 to 1.9 (Male: Female) (27, 29, 30, 32, 33). The most common site of origin was hard palate/retromolar trigone in our series and other papers (5, 27, 30, 31). This can be explained by densely populated minor salivary gland in the hard palate of the oral cavity (28). The majority of tumors (39.7%) in this study were mucoepidermoid carcinoma, followed by adenoid cystic carcinoma and adenocarcinoma. This was consistent with some studies (23, 27, 32), while others reported adenoid cystic carcinomas was the most common histological type (5, 6, 31). According to our results, most tumors were early T (T1–2) and N0 status at the time of diagnosis. Low frequency of nodal metastasis was also line with other studies even though the dominant T status was slightly different across studies (5, 6, 23, 26).

As surgery has been the primary treatment option for resectable SGC and radiation is the main adjuvant therapy for tumors with high-risk factors (34, 35), surgery with adjuvant radiation treatments was the most frequently used modality in many studies including the present paper (24, 35, 36). Despite frozen section analysis of the resection margin during surgery, 21.3% of the patients in this study had a positive resection margin (presence of cancer cells at the resection margin) and other studies have reported the rates ranging from 3.4 to 40% (5, 6, 12, 37). After treatment, more than half of the patients remained cancer free. In our series, 7.4% of patients had local recurrence while 5.9 and 27.9% of patients experienced regional and distant metastasis, respectively (Tables 1 and 4). Because of the low incidence and diversity of SGC, there are some differences in reported statistics for recurrence. Garden et al. reported 12% local recurrence and 27% distant metastases. For regional recurrence, 3 of 13 patients with initially node-positive disease had regional failure, while <5% of patient with node-negative disease had regional failure (34). Strick et al. reported 14.3% local recurrence and 33.3% distant metastases (38).

Even with some discrepancies in loco-regional outcomes, most studies indicated relatively high occurrence of distant metastasis compared to loco-regional recurrence. This is in contrast to oral SCC, which has a higher rate of loco-regional recurrence than isolated distant metastasis (39, 40). This result can be partly explained by effective suppressive role of radiation in loco-regional control of oral SGC. In 224 patients with minor salivary gland cancer, Spiro et al. reported a local failure rate of 47% after initial treatment, of which more than 90% was surgery alone (41). Weber et al. reported a local failure rate of 35% in patients with submandibular gland tumors with surgery alone, while patients with postoperative radiation showed a 15% local failure rate (42).

As tumor (T) status and subsites can affect local biological and clinical outcomes in oral cancer (1, 5, 12, 34), a propensity matching analysis was performed on these two variables in SGC and SCC groups. In a propensity-matched cohort with close resection margin, radiation was mainly used for adjuvant therapy of SGC and adjuvant chemoradiation was exclusively used in SCC. Adjuvant radiation successfully achieved 76.9–100% local control and >90% regional control in oral SGC with a >5 mm or positive resection margin (Table 5). Since SGCs are composed of tumors with various grades, only high-grade SGC was analyzed to determine whether this excellent local control of adjuvant radiation was observed in high-grade SGC. Also, we confirmed good loco-regional control by adjuvant radiation in a subgroup of high-grade SGC in the oral cavity (Table 7). Our finding was consistent with other reports (42, 43); meanwhile one recent study indicated that postoperative radiotherapy was not a statistically significant variable for overall survival in minor salivary gland cancer of the head and neck (HR, 0.64; 95% CI, 0.39-1.03, *P* = 0.068) (44). However, only 37.8% of patients had postoperative radiation in this report, which suggested somewhat a different treatment strategy (wider surgery) from our series (cases with a clear, negative margin = 48.3%). This point should be further validated through future studies.

In overall survival rate for the initial cohort, the survival rate of oral SCC was lower than that of oral SGC. Strick et al. reported that patients with SGC tend to have late recurrence with a 10- year survival of only 40% (38). However, Garden et al. showed a 5-year survival rate of 81%, a 10-year survival rate of 65% even with metastases within 5 years and late local failure events after 5 years (34). This was similar to clinical courses in our series and the survival difference between the two cancer types remained similar as the initial cohort after matching. The 5-, 10-, 15-year overall survival rate in SGC were 91.9, 72.9, and 54.7% in our series, which were comparable with other studies (78–94% at 5 years, 40–84% at 10 years, 43–73% at 15 years) (34, 38, 43, 44).

Excellent local control of oral SGC even with marginal surgical resection of the primary tumor in the oral cavity might be due to less aggressive behavior at the primary site, in addition to the effective role of adjuvant radiation treatments. Next, we examined microscopic extension from the gross tumor border in SGC and SCC. In SGC, a pushing border was more prevalent, whereas an infiltrative border occupied the majority of SCC at 87.2%. These results are consistent with previous studies reporting slow growth of SGC (24). Thus, these pathology findings can be one reason explaining the good local control in oral SGC, even with a higher rate of close or positive resection margin, compared to SCC. More interestingly, high grade SGC and adenoid cystic carcinomas had infiltrative pattern of local tumor growth (71.4%, data not shown) in our series. This emphasize that multimodal treatments (surgery with radiation) can yield a better local control in a subset of oral SGC with locally invasive features (43).

In our paper, despite the rarity and heterogeneity of SGC, we suggest a comparative overview that can be applied in management of SGC arising from the oral cavity, using a propensity score-matching and stratification according to tumor grade. However, there are some limitations to our study. The number of patients was insufficient to extrapolate our results to patients with minor pathology in SGC. Also, the results were driven from a single institution; our cohort may be under-representative of the whole SGC patients. Furthermore, since it was a retrospective study, cases with limited information were excluded or omitted from the analysis. These limitations can be solved through future studies such as multi-center research.

Compared with oral SCC, the disease course of salivary gland cancer is more indolent, slow-progressing, resulting in longer patient survival. Thus, it seems possible to adjust treatments (extent and intensity of treatments) based on the tumor biology (indolent disease course and natural history, pathology and tumor grade), which is different from oral SCC.

## Conclusion

In this study, we provided a comparative overview of clinical courses of oral SGC and SCC by a propensity-score matching analysis. To summarize, we confirmed that SGC in the oral cavity represented relatively good prognosis. A surgery with adjuvant radiation was very effective to control minimal residual disease in oral SGC, which had a locally less aggressive pathology compared with oral SCC. Our study proposed that a different treatment strategy for oral SGC based on tumor biology (pathology and tumor grade) would be reasonable in comparison with oral SCC.

## Data Availability Statement

All datasets generated for this study are included in the article/Supplementary Material.

## Ethics Statement

The studies involving human participants were reviewed and approved by The Institutional Review Board of Samsung Medical Center. Written informed consent for participation was not required for this study in accordance with the national legislation and the institutional requirements.

## Author Contributions

SP wrote the draft of the manuscript and evaluated patient record. WP and SC evaluated patient records. YJ, HK, and S-HK performed pathological investigation. JN conducted an analysis of radiation treatment. MC, Y-IS, and C-HB supervised the study and participated in quality control of data. H-SJ conceived the study concept and supervised the project and wrote and edited the manuscript. All authors read and approved the final manuscript.

## Conflict of Interest

The authors declare that the research was conducted in the absence of any commercial or financial relationships that could be construed as a potential conflict of interest.

## Supplementary Material

Supplementary Data Sheet 1 The raw dataset.
